# Spinal cord swelling in patients with cervical compression myelopathy

**DOI:** 10.1186/s12891-019-2673-2

**Published:** 2019-06-14

**Authors:** Naohiro Tachibana, Takeshi Oichi, So Kato, Yusuke Sato, Hiroyuki Hasebe, Shima Hirai, Yuki Taniguchi, Yoshitaka Matsubayashi, Harushi Mori, Sakae Tanaka, Yasushi Oshima

**Affiliations:** 1 Department of Orthopaedic Surgery, Faculty of Medicine, The University of Tokyo, 7-3-1 Hongo, Bunkyo-ku, Tokyo, 113-0033 Japan; 2 Department of Radiology, Faculty of Medicine, The University of Tokyo, Tokyo, Japan

**Keywords:** Spinal cord edema, Cervical spondylosis, Intramedullary hyperintense lesion, Early-onset, Rapid disease progression

## Abstract

**Background:**

Intramedullary hyperintense lesions associated with spinal cord edema on T2-weighted MR images (T2WI) are rare findings in patients with cervical spondylosis and are poorly characterized. We investigated the clinical characteristics of spinal cord edema due to cervical spondylosis (SCECS).

**Methods:**

In total, 214 patients with cervical spondylotic myelopathy who underwent surgery between April 2007 and March 2017 were divided into SCECS and non-SCECS groups with SCECS defined as follows: (1) intramedullary signal intensity (ISI) of the cervical spinal cord in sagittal T2WI extending to more than one vertebral body height; (2) “fuzzy” ISI, recognized as a faint intramedullary change with a largely indistinct and hazy border; and (3) a larger sagittal diameter of the spinal cord segment with ISI just above or below the cord compression area compared with areas of the cervical spine without ISI. Radiographic parameters, demographic characteristics, and the Japanese Orthopedic Association (JOA) surgical outcomes score were compared between the groups.

**Results:**

Seventeen patients (7.9%) were diagnosed with SCECS. These patients were younger than those in the non-SCECS group [median (interquartile range), 64 (20) vs. 69 (15) years, respectively, *p* = 0.016], and the disease duration from onset to surgery was significantly shorter in the SCECS group than in the non-SCECS group [6 (7) vs. 20 (48) months, respectively]. No significant difference was observed between groups with respect to sex, radiologic findings, or surgical outcomes.

**Conclusion:**

The disease showed an earlier onset and more rapid progression in the patients with SCECS than in those without SCECS.

## Background

Most intramedullary lesions in cervical compressive myelopathy are gray matter myelomalacia and present with a snake-eye appearance on T2-weighted MR images (T2WI). Zhou et al. described myelomalacia as a radiographical finding on MRI manifested by an ill-defined area of cord signal change visible on T1- and T2-weighted sequences as hypo- and hyperintense areas and commonly associated with focal cord atrophy [1]. Based on autopsy results, Mizuno et al. suggested that the snake-eye appearance was a result of cystic necrosis resulting from mechanical compression and venous infarction [2].

Intramedullary hyperintense lesions associated with spinal cord edema on T2WI are rare findings in patients with cervical spondylosis. In a report of six patients with cervical spinal cord edema, Lee et al. speculated that the radiological characterization of spinal cord edema was based on reversible white matter lesions, which were most likely caused by disturbed local venous circulation [3]. Such edematous lesions are liable to be misinterpreted as neoplastic or inflammatory lesions in the spinal canal, and this can delay appropriate treatment. However, intramedullary hyperintense lesions with spinal cord edema are not well characterized in the literature. The purpose of this study was to investigate the clinical characteristics of spinal cord edema due to cervical spondylosis (SCECS).

## Methods

### Subjects

This retrospective cohort study included 214 patients with cervical spondylotic myelopathy who underwent surgery between April 2007 and March 2017 at our institution. Patients with disc hernia, ossification of the longitudinal ligament, tumors, rheumatoid arthritis, or a history of trauma and spine surgery were excluded. The selection of the surgeon, patients, and operative methods was not randomized.

We divided the patients into two groups, SCECS group and non-SCECS group. Radiographic parameters, demographic characteristics of patients, and surgical outcomes characterized based on the Japanese Orthopedic Association (JOA) score were compared between these two groups.

For further analysis, we divided the patients in the non-SCECS group into three subgroups comprising those negative for ISI on MRI, those positive for ISI only on T2WI, and those positive for ISI on both T1-weighted MR images (T1WI) and T2WI. We compared these three groups and the SCECS group with respect to the same factors as in the initial analysis.

### Measurements using radiographs and MRI

Cervical spine radiographs were used to measure the diameter of the spinal canal, C2–7 cervical lordosis (CL), C2–7 range of motion, and segmental range of motion and dynamic instability at the level of greatest stenosis. The definition of dynamic instability was an anterior vertebral slip of ≥3.5 mm or an increase in vertebral angulation in flexion of ≥11°, as previously reported [4].

We defined SCECS as follows: (1) intramedullary signal intensity (ISI) of the cervical spinal cord in sagittal T2WI extending to more than one vertebral body height; (2) “fuzzy” ISI, recognized as a faint intramedullary change with a largely indistinct and hazy border; and (3) a larger sagittal diameter of the spinal cord segment with ISI just above or below the cord compression area compared with areas of the cervical spine without ISI.

### Statistical analysis

The Mann–Whitney U test was used for continuous outcomes and the χ^2^ test and Fisher’s exact test were used for binomial outcomes. The Kruskal–Wallis test with Bonferroni adjustment was used for multiple comparisons. A *p* value less than 0.05 was considered statistically significant. The analyses were carried out using IBM SPSS Statistics version 19 (IBM Corp., Armonk, New York, United States).

## Results

Of the 214 patients with cervical spondylotic myelopathy who underwent surgery, 17 (7.9%) were diagnosed with SCECS and assigned to the SCECS group. The non-SCECS group therefore included 197 patients. Of the SCECS group patients, 16 underwent laminoplasty and one underwent anterior cervical discectomy and fusion (ACDF). In the non-SCECS group, 168 patients underwent laminoplasty, 11 underwent pedicle screw fixation, and 18 underwent ACDF. The patients in the SCECS group were younger than those in the non-SCECS group [median (interquartile range), 64 (20) vs. 69 (15) years, respectively, *p* = 0.016] (Table 1). The median time from the onset of the disease to surgery was significantly shorter in the SCECS group than in the non-SCECS group [6 (7) vs. 20 (48) months, respectively]. However, no significant between-group differences were observed with respect to sex, preoperative radiologic findings, preoperative JOA score, postoperative JOA score, or JOA recovery rate.

**Table 1:** Patient characteristics compared between the groups with and without spinal cord edema due to cervical spondylosis (SCECS)

	SCECS (*n* = 17)	Non-SCECS (*n* = 197)	*p* value
Age (years) *1	64 (20)	69 (15)	*0.016
Male (%) *2	15 (88)	135 (69)	0.089
BMI *1	25.4 (5.3)	23.9 (5.1)	*0.038
Smoking (%) *2	6 (35)	76 (39)	1
Time from onset to operation (months) *1	6 (7)	20 (48)	* < 0.001
Postoperative follow-up period (months) *1	24 (53)	27 (41)	0.57
Preoperative C2–7 cervical lordosis (°) *1	13 (17)	13 (14)	0.502
Preoperative dynamic instability (%) *3	1 (5.8)	21 (10.6)	0.455
Preoperative C2–7 range of motion (degrees) *1	35 (13)	37 (20)	0.399
Preoperative segmental range of motion (°) *1	10 (8)	8 (6)	0.516
Diameter of the spinal canal (mm) *1	14 (2)	14 (2)	0.913
Preoperative JOA total score *1	10.0 (4.0)	11.0 (3.0)	0.573
Postoperative JOA total score *1	13.0 (3.5)	12.0 (3)	0.887
JOA recovery rate (%) *1	37.5 (28.5)	28.6 (35.7)	0.361
Reoperation (%) *3	0 (0)	10 (10.3)	0.427

Data are presented as median (interquartile range) or frequencies (%)

*1 Mann–Whitney U test, *2 χ2 test, *3 Fisher’s exact test

In comparisons involving the SCECS group and the three non-SCECS subgroups (those negative for ISI on MRI, positive for ISI only on T2WI, or positive for ISI on both T1WI and T2WI), the patients in the SCECS group were significantly younger than those in the other groups (Table 2). However, no significant between-group differences were observed with respect to sex, time from disease onset to surgery, preoperative radiologic findings, preoperative JOA score, postoperative JOA, or JOA recovery rate.

**Table 2:** Comparison of characteristics between the patients with spinal cord edema due to cervical spondylosis (SCECS) group and the three non-SCECS subgroups [those negative for intramedullary signal intensity (ISI) on MRI; those positive for ISI on only T2-weighted MR images (T2WI); and those positive for ISI on both T1- and T2-weighted MR images (T1 & T2WI)]

	SCECS (*n* = 17)	Negative ISI (*n* = 82)	ISI on T2WI (*n* = 96)	ISI on T1/T2WI (n = 19)	*p* value
Age (years) *1	*64 (20)	*73 (14)	67 (14)	68 (17)	*0.032
Male (%) *2	15 (88)	53 (65)	67 (70)	14 (74)	0.239
BMI (kg/m^2^) *1	25.4 (5.3)	23.6 (4.4)	23.6 (5.2)	26.2 (5)	0.08
Smoking (%) *2	6 (35)	30 (37)	36 (38)	10 (53)	1
Time from onset to operation (months) *1	6 (7)	21 (50)	19 (47)	31 (45)	0.129
Postoperative follow-up period (months) *1	24 (53)	24 (32)	37 (44)	27 (45)	0.098
Preoperative C2–7 cervical lordosis (°) *1	13 (17)	14.5 (13)	13 (13)	5.5 (19)	0.11
Preoperative C2–7 range of motion (°) *1	35 (13)	36.5 (21)	36.5 (18)	42 (21)	0.51
Preoperative segmental range of motion (°) *1	10 (8)	7 (7)	8.5 (6)	7 (6)	0.323
Diameter of the spinal canal (mm) *1	14 (2)	*14 (2)	*14 (1)	14 (1)	*0.036
Preoperative JOA total score *1	10.0 (4.0)	10.8 (3.0)	11.0 (3.0)	11.0 (2.3)	0.938
Postoperative JOA total score *1	13.0 (3.5)	12.0 (3.0)	13.0 (4.0)	12.0 (3.3)	0.754
JOA recovery rate (%) *1	37.5 (28.5)	25.0 (33.0)	33.3 (42.2)	26.8 (41.7)	0.499
Reoperation (%) *2	0 (0)	7 (8.5)	3 (3.2)	0 (0)	1

Data are presented as median (interquartile range) or frequencies (%)

*1 Kruskal–Wallis test and Bonferroni adjustment, *2 Fisher’s exact test

BMI: body mass index; JOA: Japanese Orthopedic Association surgical outcome score

**Fig. 1:**
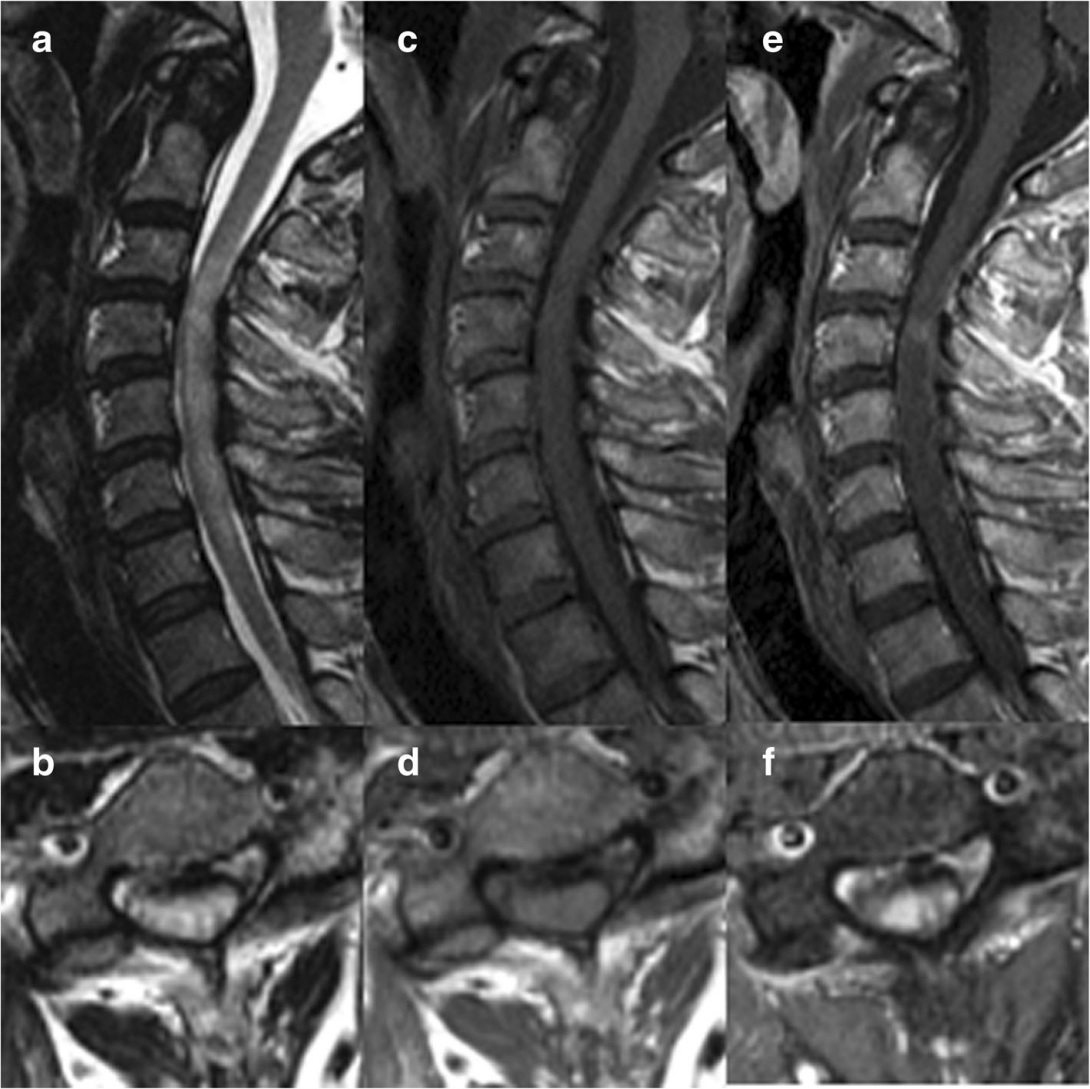
MRI images of a 53-year-old man with spinal cord edema due to cervical spondylosis revealing T2 high (**a**, **b**), T1 iso (**c**, **d**) signal intensity, and pancake-like gadolinium enhancement (**e**, **f**)

We investigated the relevance of preoperative Gd-enhanced MRI and postoperative ISI regression to surgical outcomes in the SCECS group. Of the 17 patients with SCECS, 11 underwent preoperative Gd-enhanced MRI; Gd enhancement was observed in six patients (55%). Three patients exhibited a pancake-like (i.e., flat and roughly circular) transverse band of Gd enhancement (Fig. 1) [5]. However, the analysis showed that Gd enhancement did not affect the surgical outcomes (Table 3).

**Table 3:** Comparison between the patients positive and negative for gadolinium (Gd) enhancement among those with spinal cord edema due to cervical spondylosis

	Gd enhancement (+) (*n* = 6)	Gd enhancement (−) (*n* = 5)	p value
Age (y) *1	63 (31)	57 (20)	0.855
Male (%) *2	4 (66)	5 (100)	0.455
Time from onset to operation (months) *1	9 (10)	5 (3)	0.195
Postoperative follow-up period (months) *1	18.5 (29)	63 (41)	0.01
Preoperative C2–7 range of motion (°) *1	21.5 (20)	32 (13)	0.2
Preoperative segmental range of motion (°) *1	11.5 (6)	4 (8)	0.054
Diameter of the spinal canal (mm) *1	14.5 (1)	14 (3)	0.099
Preoperative JOA total score *1	10.25 (6.3)	12 (4.3)	0.199
Postoperative JOA total score *1	11.5 (4.5)	15 (2.5)	0.139
JOA recovery rate(%) *1	25.8 (22.7)	50 (53.3)	1

Data are presented as median (interquartile range) or frequencies (%)

*1 Mann–Whitney U test, *2 χ2 test

JOA: Japanese Orthopedic Association surgical outcome score

Postoperatively, all patients in the SCECS group underwent MRI two weeks after surgery and six of them (35%) exhibited ISI regression. Of the 11 patients who did not show ISI regression two weeks after surgery, five patients underwent another MRI at final follow-up (1–6 years after surgery), four of whom exhibited ISI regression. In total, 10 (59%) patients in the SCECS group exhibited ISI regression. There was no significant correlation between the postoperative ISI regression two weeks after surgery and surgical outcomes (Table 4).

**Table 4:** Comparison between the patients with and without intramedullary signal intensity (ISI) regression among those with spinal cord edema due to cervical spondylosis

	ISI regression (+) (*n* = 6)	ISI regression (−) (*n* = 11)	*p* value
Age (years) *1	67.5 (21)	58 (23)	0.84
Male (%) *2	6 (100)	9 (82)	0.52
Time from onset to operation (months) *1	7 (11)	6 (5)	0.477
Postoperative follow-up period (months) *1	18.5 (44)	24 (58)	0.801
Preoperative C2–7 range of motion (°) *1	33.5 (19)	36 (12)	0.546
Preoperative segmental range of motion (°) *1	10.5 (6)	9 (8)	0.338
Diameter of the spinal canal (mm) *1	14 (2)	14 (2)	0.372
Preoperative JOA total score *1	11 (3.9)	9 (4)	0.363
Postoperative JOA total score *1	14 (4.4)	12 (2)	0.263
JOA recovery rate (%) *1	46.5 (31.6)	30.0 (20.0)	0.078

Data are presented as median (interquartile range) or frequencies (%)

*1 Mann–Whitney U test, *2 χ2 test

JOA: Japanese Orthopedic Association surgical outcome score

## Discussion

### The pathophysiology of spinal cord swelling

In the present study, the patients with SCECS were younger and they showed significantly shorter duration of the disease than those without SCECS. We speculated that the pathology in those with SCECS was different from that in the typical degenerative myelopathy cases. Although the pathophysiology of spinal cord swelling has been poorly understood, involvement of venous hypertension of the spinal cord has been indicated. Lee et al. speculated that impaired venous return due to spinal cord compression causes local venous hypertension, which leads to venous ischemia and spinal cord edema at the compression site or adjacent levels [3]. Other authors have suggested that disturbed cerebrospinal fluid (CSF) circulation may play a role in the development of spinal cord edema [6, 7]. Okada et al. reported a case of venous hypertensive myelopathy associated with cervical spondylosis and hypothesized that cervical canal stenosis and cord compression impaired the spinal venous system, resulting in progressive myelopathy [8]. In addition, mechanical stress by neck motion might be related to the onset of spinal cord swelling, which is still controversial. Sasamori et al. reported that transient but repetitive cord compression was associated with spinal cord swelling and Gd enhancement [9]. Hattou et al. described six young cervical myelopathy patients with non-traumatic cervical chronic joint instability [10].

**Fig. 2:**
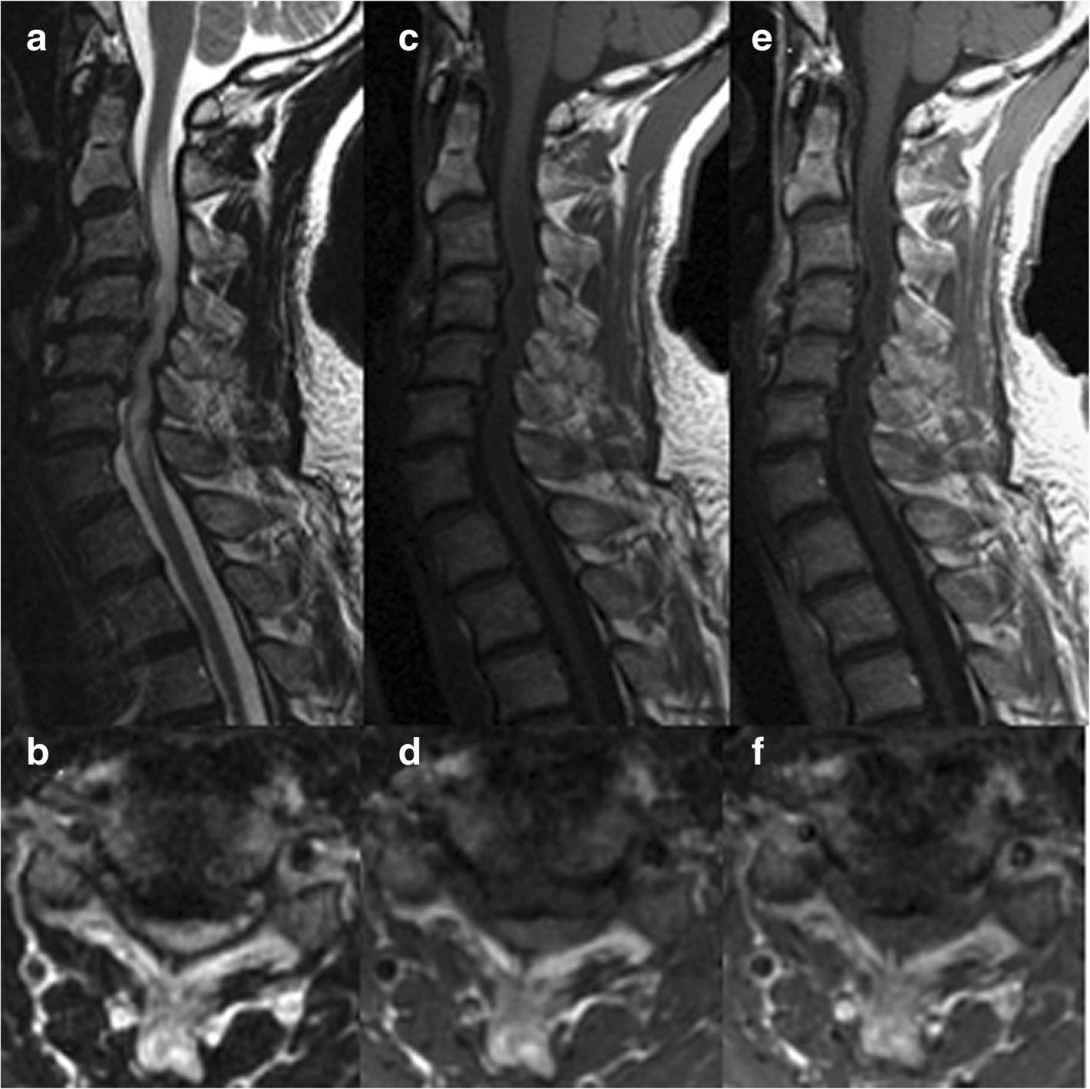
MRI images of a 67-year-old man with spinal cord edema due to cervical spondylosis revealing T2 high (**a**, **b**), T1 iso (**c**, **d**) signal intensity, and negative gadolinium enhancement (**e**, **f**)

In addition, six of the 17 patients with SCECS in our study exhibited Gd enhancement (Figs. 1,2). This was a much higher proportion than that found in previous retrospective and prospective studies, which reported Gd enhancement in 7–10% of patients with cervical spondylotic myelopathy [11–13]. According to the past reports, a breach of the blood-spinal cord barrier (BSCS) of the white matter vessels could be related to the positive intramedullary Gd enhancement, which results in important pathological changes, including cytotoxic edema and vasogenic edema, which were frequently associated with aquaporins (water channel proteins) [3, 14–16]. Aquaporins in the central nervous system (CNS) have mainly been studied with evidence that they play important roles in the pathogenesis of CNS injury, edema, and various diseases such as multiple sclerosis, neuromyelitis optica spectrum disorders, and glioblastoma multiforme [14, 17, 18]. The MRI findings in SCECS resemble the findings in the above pathologies, which indicates the underlying pathophysiological mechanisms are similar.

Based on our findings of earlier onset, more rapid progression, and high proportion of Gd enhancement, we speculate that the pathogenesis of SCECS was triggered by disturbed venous circulation and the changes in the expression of aquaporins.

### Surgical outcomes

Unlike previous studies [3, 5, 19], we investigated the differences in surgical outcomes between the SCECS group and non-SCECS subgroups. Various studies have investigated the prognostic significance of ISI on T1WI, T2WI, and Gd enhancement [20–22], but the relevance of these imaging findings to clinical outcome remains controversial.

In the present study, there was no significant difference between the SCECS group and non-SCECS subgroups with respect to surgical outcome. Further, there was no significant correlation between Gd enhancement, postoperative ISI regression, and surgical outcomes. In the study by Lee et al., five of six patients exhibiting ISI regression experienced good improvement of symptoms, although spinal cord edema observed during the follow-up MR imaging persisted for several months after surgery [3]. Flanagan et al. reported that 53 (95%) of 56 patients with Gd enhancement were stable or improved with Gd enhancement persisting at 12 months in 42 (75%) patients [5]. Lee et al. speculated that the healing process following hyperlucency or a break in the BSCB is long-standing and that the spinal cord recovering its previous function and decompression causes acute reduction of the intravascular resistance, inducing in postoperative aggravation of spinal cord swelling [3]. In our study, six of 17 (35%) patients exhibited ISI regression at an early stage after surgery. Further, excluding six patients who exhibited ISI regression at an early stage after surgery, four of 11 (36%) exhibited ISI regression at the final follow-up. We speculate that SCECS has reversible and unstable features. Although this disease did not affect the surgical outcome, the lack of between-group differences may have been due to the small sample size.

### Diagnosis and management

**Fig. 3:**
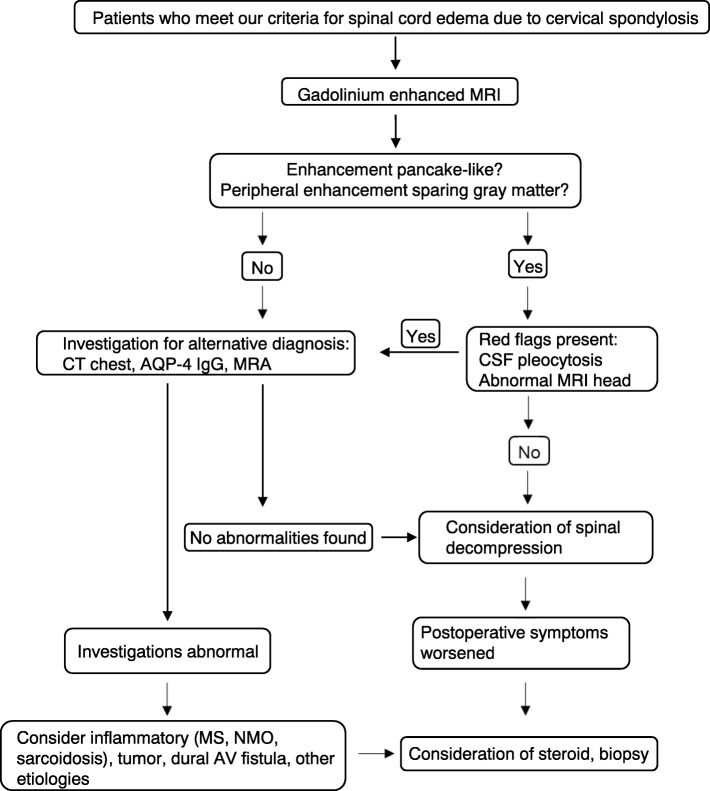
Algorithm for management of spinal cord edema due to cervical spondylosis, modified from that of Flanagan et al. [5]. AQP4 Aquaporin-4; *AV* Arteriovenous, *CSF* Cerebrospinal fluid, *CT* Computed tomography, *IgG* Immunoglobulin G, *MRA* Magnetic resonance angiography; *MRI* Magnetic resonance imaging, *MS* Multiple sclerosis, *NMO* Neuromyelitis optica

In the present study, none of the patients with SCECS received an alternative diagnosis postoperatively. Nurboja et al. reported the case of a patient who failed to improve postoperatively and was found to have neurosarcoidosis [19]. Flanagan et al. reported that 40 (71%) of their patients had initially been diagnosed with neoplastic or inflammatory myelopathies with decompressive surgery delayed by a median of 11 months [5]. They recommended that patients who present with T2-high signal changes and contrast enhancement should be closely investigated using brain MRI and CSF, hematological, and biochemical analyses [5, 19]. We agree with their recommendation; however, most patients with cervical spondylotic myelopathy undergo plain MRI in the clinical setting. Although it was not possible to investigate all the patients with SCECS using Gd-enhanced MRI because of the retrospective nature of this study, we recommend that patients who meet our definition of SCECS should be considered for additional investigations including Gd-enhanced MRI and referred to neurologists (Fig. 3). In our opinion, surgeons should inform patients with SCECS about possible alternative pathologies including tumors, inflammatory disorders before surgery and follow this up postoperatively. In our study, no patient underwent spinal cord biopsy. Flanagan et al. reported the cases of six patients who underwent biopsies which did not reveal any alternative diagnoses. (5) Nurboja et al. proposed that in the first instance, spinal cord decompression should be considered rather than biopsy when no neurological cause is found [19]. Cohen-Gadol et al. reported that specific treatment was determined based on spinal cord biopsy results in only 26% of patients [23]. They also reported a high (21%) complication rate for spinal cord biopsy, including neurological deficits. Furthermore, there was no significant difference between the SCECS group and non-SCECS subgroups with respect to surgical outcomes in our study. We therefore recommend that spinal cord biopsy should be considered only when sarcoidosis or intramurally tumors are highly suspected or when postoperative symptoms deteriorate.

### Limitations

The present study had several limitations. First, it was a retrospective study. The follow-up period was relatively short, and the sample size was small. Second, not all patients underwent Gd-enhanced MRI or a thorough investigation to rule out sarcoidosis. Third, there were few data of MRI at the final follow-up. Finally, the patient-reported outcomes were not evaluated. Although differences in the JOA scores were not significant, the pain levels or the patient’s quality of life may have been affected. Further studies are required to clarify the pathophysiology and more detailed surgical outcomes of the SCECS treatment.

## Conclusions

The incidence of SCECS was 7.9% in this study. We found that patients with SCECS were significantly younger than those without SCECS and that the time from the onset of the disease to surgery in these patients was significantly shorter; however, surgical outcomes were similar.

## Data Availability

The datasets suporting the conclusions of this article are included within the article. The raw data underlying the conclusions made in this study can be inquired to the first author.

## Abbreviations

ACDF: Anterior cervical discectomy and fusion
BSCB: Blood-spinal cord barrier
CL: Cervical lordosis
CNS: Central nervous system
CSF: Cerebrospinal fluid
ISI: Intramedullary signal intensity
JOA: Japanese Orthopedic Association
SCECS: Spinal cord edema due to cervical spondylosis
T1WI: T1-weighted MR images
T2WI: T2-weighted MR images
